# Phytochemical analysis with free radical scavenging, nitric oxide inhibition and antiproliferative activity of *Sarcocephalus pobeguinii* extracts

**DOI:** 10.1186/s12906-017-1712-5

**Published:** 2017-04-04

**Authors:** Emmanuel Mfotie Njoya, Aristide Mfifen Munvera, Pierre Mkounga, Augustin Ephrem Nkengfack, Lyndy Joy McGaw

**Affiliations:** 1 Department of Paraclinical Sciences, Faculty of Veterinary Science, University of Pretoria, Private Bag X04, Onderstepoort, Pretoria, 0110 South Africa; 2 Department of Biochemistry, Faculty of Science, University of Yaoundé I, P.O. Box 812, Yaoundé, Cameroon; 3 Department of Organic Chemistry, Faculty of Science, University of Yaoundé I, P.O. Box 812, Yaoundé, Cameroon

**Keywords:** *Sarcocephalus pobeguinii*, Antioxidant, Anti-inflammatory, Cancer, Selective cytotoxicity

## Abstract

**Background:**

Free radicals have been implicated in the pathogenesis of diverse metabolic disorders including cancer. Therefore, fighting against free radicals has become an important strategy in the prevention or treatment of such diseases, in addition to direct or indirect anticancer chemotherapy. *Sarcocephalus pobeguinii* has been used traditionally to treat various diseases in which excess production of free radicals is implicated, warranting investigation of its free radical scavenging, anticancer and anti-inflammatory activity.

**Methods:**

In the present study, extracts from leaves, fruits, roots and bark of *Sarcocephalus pobeguinii* were evaluated on four human cancer cell lines (MCF-7, HeLa, Caco-2 and A549 cells) and a non-cancerous cell line for their antiproliferative potential. The cells were incubated with the plant extracts for 48 h at 37 °C in a 5% CO_2_ humidified environment and their cytotoxic effect was determined using the tetrazolium-based colorimetric (MTT) assay. The radical inhibition was determined using the 2,2-diphenyl-1-picrylhydrazyl (DPPH) and the 2,2′-azino-bis (3-ethylbenzothiazoline-6-sulfonic acid) (ABTS) scavenging techniques. The nitric oxide inhibitory activity was determined using LPS-activated RAW 264.7 macrophages. The correlation between radical scavenging capacity and antiproliferative activity was also analysed.

**Results:**

The extract from leaves of *Sarcocephalus pobeguinii* (LSP) exhibited the highest cytotoxic effect on all four of the human cancer cell lines but with some cytotoxicity to the normal Vero cells. However, the LSP extract had the best selectivity index, ranging from 3.15 to 18.28. Also, antioxidant and anti-inflammatory assays indicated that the LSP extract had the highest radical scavenging capacity of all the extracts. A positive linear correlation was found between free radical scavenging ability and antiproliferative activity against the four cancer cell lines, with the highest correlation factor (R^2^ = 0.9914) obtained between DPPH inhibition and antiproliferative activity against A549 cells.

**Conclusions:**

The high selectivity index of the *Sarcocephalus pobeguinii* leaf extract indicates the potential of using this extract in cancer therapy. Furthermore, the positive correlation between free radical scavenging and antiproliferative activity suggests that the radical scavenging capacity of extracts may contribute to a prediction of their anticancer property.

## Background

Plants are susceptible to environmental stress and have developed numerous defense systems, resulting in formation of potent antioxidants. By definition, antioxidants are complex compounds found in our diet that act as a protective shield for our body against certain disastrous diseases [1]. Many biological functions, such as protection from mutagenesis, carcinogenesis, inflammation and ageing are due to antioxidative effects. Oxidative stress has emerged as one of the important factors in the pathogenesis of cancer and age-related disorders. Cancer represents a major public health problem around the world with approximately 8.4 million deaths in 2012 [2]. Environmental, chemical, metabolic and genetic factors play a direct or indirect role in the development of cancer. Amongst these factors, free radicals like hydroxyl, peroxyl and superoxide radicals appear to be one of the fundamental mechanisms explaining the initiation and progression of cancer. In fact, free radicals can bind through electron pairing with biological macromolecules such as proteins, lipids and DNA in healthy human cells and cause protein and DNA damage along with lipid peroxidation [3]. Furthermore, oxidative DNA damage is a major cause of mutation, and the relevance of DNA damage in the progression of cancer (carcinogenesis) is quite established. The accumulation of such genetic events in normal cell lines initiates a progressively dysplastic cellular appearance, which leads to deregulated cell growth, and finally carcinoma [4]. The treatment of cancer includes the application of chemotherapy, radiotherapy or surgical intervention. In some cases, due to drug resistance or late medical intervention, some surviving cancer cells escape from such treatment and continue their proliferation. Therefore, it is urgent to search for new strategies to fight cancer.

Medicinal plants represent alternative prophylactic and therapeutic measures which can be efficiently used to control various diseases. Medicinal plants have been used empirically as drugs, initially as traditional preparations and then as pure active principles, with knowledge and practice passing from generation to generation [5]. Based on their diverse composition of various secondary metabolites, medicinal plants are used to prevent or cure disease, or to promote general health and well-being [6]**.** *Sarcocephalus pobeguinii* (Hua ex Pobég) (synonym of *Nauclea pobeguinii* (Hua ex Pobég) Merr.) is a shrub of the Rubiaceae family widespread in sub-Saharan Africa and is used in the treatment of various ailments such as gonorrhoea, malaria, stomach-ache, threatened abortion, epilepsy, pain, fever, infectious diseases and jaundice [7]. The ethanolic extract of *Sarcocephalus pobeguinii* has been reported to exhibit good antimalarial activity in vitro against *Plasmodium falciparum* [8] and in vivo against *Plasmodium berghei* and *Plasmodium yoellii* mice models [9]. Clinical trials of an herbal medicine prepared with the ethanolic extract of the stem bark of *S. pobeguinii* indicated the safety and efficacy of this herbal medicine in the treatment of uncomplicated malaria [10–12]. Extracts and some isolated compounds from the bark of *S. pobeguinii* were identified with antiproliferative potential against drug-resistant cancer cell lines [13]. Therefore, our aim in the present study is to evaluate the selective cytotoxicity of different extracts of this species on other cancer cell lines and determine the correlation between the radical scavenging capacity of active extracts and their antiproliferative activity.

## Methods

### Collection and preparation of plant extracts

Roots, fruits, bark and leaves of *Sarcocephalus pobeguinii* were collected in Ezezan (Nyom II), a locality situated 40 km from Yaoundé (Cameroon). A voucher specimen was prepared and identification was made at the National Herbarium of Cameroon by comparison with the number N°32567 BRF/CAM and a sample deposit was registered under the number Letouzey R.12493 (YA). After drying at room temperature, the collected samples were ground and the different powders obtained were used for extraction. The fruits were steeped in CH_2_Cl_2_/MeOH (1:2) for 48 h at room temperature while roots, leaves and bark were extracted in methanol and heated at 60–70 °C for 2 h. Each mixture was filtered with Whatman No.1 filter paper and the filtrate was dried using a rotavapor (RV10 Basic, IKA) to obtain a residue which constituted the crude extract. The yield of extraction of each type of plant material is indicated in Table 1.

**Table 1:** Percentage yield of extraction and phytochemical composition of different extracts

Plant material	Yield of crude extract (%)	Phytochemical content					
		Saponins	Alkaloids	Phenolics	Flavonoids	Terpenoids	Tanins
Roots	10.733	---	--+	---	---	+++	+++
Fruits	8.066	+++	-++	---	---	+++	+++
Bark	28.400	+++	+++	--+	+++	+++	+++
Leaves	11.733	+++	+++	-- +	+++	+++	+++

(+) indicates presence and the intensity of color or precipitate formation while (-) means the chemical is not detected

### Phytochemical screening

The phytochemical content of extracts was evaluated according to the methods described by Sofowora [14] and Trease and Evans [15].

#### Test for alkaloids

One gram (1 g) of each powder was stirred with 5 mL of 1% aqueous HCl in a water bath and then filtered. One milliliter (1 mL) of the filtrate was aliquoted individually into 2 test tubes. To the first portion, a few drops of Dragendorff’s reagent were added and the occurrence of an orange-red precipitate was taken as a positive reaction for alkaloids. To the second portion, 1 mL of Mayer’s reagent was added and the appearance of a buff-coloured precipitate indicated the presence of alkaloids [14].

#### Test for saponins

One gram (1 g) of each powder was boiled with 5 mL of distilled water and filtered. To the filtrate, 3 mL of distilled water was further added and shaken vigorously for about 5 min. Frothing which persisted on warming was taken as evidence for the presence of saponins [14].

#### Test for terpenoids

A small quantity of each extract was dissolved in 2 mL of ethanol. To this was added 1 mL of acetic anhydride followed by the addition of concentrated H_2_SO_4_. A change in colour from pink to violet showed the presence of terpenoids [14].

#### Test for phenolic compounds

To the extract dissolved in methanol was added 3 drops of a freshly prepared ferric cyanide solution (1 mL of 1% FeCl_3_ and 1 mL of K_3_Fe (CN)). The appearance of a blue-green color indicated the presence of phenolic compounds [14].

#### Test for flavonoids

Five hundred mg of each powder was dissolved in ethanol, warmed and then filtered. Three pieces of magnesium chips were then added to the filtrate followed by a few drops of concentrated hydrochloride acid. A pink, orange, or red to purple colouration indicated the presence of flavonoids [15].

#### Test for tannins

Five hundred mg of each powder was stirred with 10 mL of distilled water and then filtered. A few drops of 1% ferric chloride solution were added to 2 mL of the filtrate. The occurrence of a blue-black, green or blue-green precipitate indicated the presence of tannins [15].

### Cell culture

All the four cancer cell lines (MCF-7: human breast adenocarcinoma cells; HeLa: human cervix adenocarcinoma cells; Caco-2: human epithelial colorectal adenocarcinoma cells; A549: human epithelial lung adenocarcinoma cells) were obtained from the American Type Culture Collection (ATCC) (Rockville, MD, USA). These cells were grown at 37 °C with 5% CO_2_ in a humidified environment in Dulbecco’s Modified Eagle’s Medium (DMEM) high glucose (4.5 g/L) containing L-glutamine (4 mM) and sodium-pyruvate (Hyclone™) supplemented with 10% (*v*/v) fetal bovine serum (Capricorn Scientific Gmbh, South America). African green monkey (Vero) kidney cell lines (also obtained from ATCC) were maintained at 37 °C and 5% CO_2_ in a humidified environment in DMEM high glucose (4.5 g/L) containing L-glutamine (Lonza, Belgium) and supplemented with 5% fetal bovine serum (Capricorn Scientific Gmbh, South America) and 1% gentamicin (Virbac, RSA). The RAW 264.7 murine macrophage cells (obtained from ATCC) were maintained in DMEM high glucose (4.5 g/L) containing L-glutamine (Lonza, Belgium) and supplemented with 10% fetal bovine serum (Capricorn Scientific Gmbh, South America) and 1% penicillin/streptomycin/fungizone (PSF) solution under 5% CO_2_ humidified environment at 37 °C.

### Cell treatment and antiproliferation assay

The cancer cells and Vero cells were inoculated at a density of 10^4^ cells per well in 96-well microtitre plates. After seeding, they were treated with increasing concentrations of the extracts dissolved in dimethyl sulfoxide (DMSO) and diluted in fresh culture medium. During each experiment, the maximal concentration of DMSO in the medium did not exceed 1%. Doxorubicin hydrochloride (Pfizer) was used as a positive control and negative controls were included. After incubation for 48 h at 37 °C with 5% CO_2_, the culture medium was discarded and replaced by fresh medium with 30 μL of thiazolyl blue tetrazolium bromide (5 mg/mL) dissolved in phosphate buffered saline. After incubation for 4 h, the medium was aspirated and the formazan crystals were dissolved in 50 μL of DMSO for 15 min. The absorbance was measured spectrophotometrically at 570 nm in a Biotek Synergy microplate reader.

The viability rate of treated cells was calculated for each concentration and the 50% inhibitory concentrations (IC_50_) for cancer cells lines and the 50% lethal concentrations (LC_50_) for normal Vero cells were determined by plotting the graph of viability rate versus the concentrations. The selectivity index (SI) values were calculated for each extract by dividing the LC_50_ of normal Vero cells by the IC_50_ of cancer cells in the same units.

### Radical inhibition assays

#### The 2,2-diphenyl-1-picrylhydrazyl (DPPH) radical scavenging assay

The DPPH scavenging capacity of extracts was evaluated using the method described by Brand-Williams et al. [16] with some modifications in microtitre format. Briefly, a methanolic solution of DPPH (160 μL) was added to samples (40 μL) and the mixture was allowed to stand at room temperature in the dark for 30 min. The absorbance of the resulting solution was measured at 517 nm using a microplate reader (Epoch, BioTek). Ascorbic acid and trolox were used as positive controls, methanol as negative control and sample without DPPH as blank. The percentage of DPPH scavenging capacity was calculated at each concentration according to the formula below:

1 $$ \mathrm{Scavenging}\kern0.5em \mathrm{capacity}\kern0.5em \left(\%\right)=100-\left[\right.\frac{\mathrm{Absorbance}\kern0.5em \left(\mathrm{sample}\right)\kern0.5em -\kern0.5em \mathrm{Absorbance}\kern0.5em \left(\mathrm{sample}\kern0.5em \mathrm{blank}\right)}{\mathrm{Absorbance}\kern0.5em \left(\mathrm{control}\right)}\times 100\left.\right] $$

The concentration of the extract leading to 50% reduction of DPPH color (IC_50_) was also determined by plotting the graph of percentage DPPH scavenging capacity against the different concentrations of the extract.

#### The 2,2′-azino-bis (3-ethylbenzothiazoline-6-sulfonic acid) (ABTS) radical scavenging assay

The ABTS radical scavenging capacity of the extracts was measured as described by Re et al. [17] with modifications to the 96-well microtitre plate. The ABTS radical was produced by reacting a solution of ABTS (7 mM) with a solution of potassium persulfate (2.45 mM) at room temperature for 12 h. The absorbance of the ABTS radical produced was adjusted to 0.70 ± 0.02 at 734 nm before use. The ABTS solution (160 μL) was mixed with the samples (40 μL) at different concentrations and the absorbance was measured after 5 min at 734 nm using a microplate reader (Epoch, BioTek). Trolox and ascorbic acid were used as positive controls, methanol as negative control and sample without ABTS as blank. The percentage of DPPH scavenging capacity was calculated at each concentration according to the formula (1) above and the IC_50_ values were calculated from the graph plotted as inhibition percentage against the concentrations.

### Nitric oxide inhibitory assay

Nitric oxide (NO) production by RAW 264.7 macrophages was determined by measuring the accumulation of nitrite, an indicator of NO in the supernatant after 24 h of lipopolysaccharide (LPS) treatment with or without the extracts or quercetin (positive control) using the Griess reagent. Briefly, the RAW 264.7 macrophages were seeded at a density of 2 × 10^4^ cells per well in 96 well-microtitre plates and the cells were allowed to attach overnight. The cells were activated by incubation in a medium containing 5 μg/mL LPS alone (control) and treated simultaneously with different concentrations of the samples dissolved in DMSO. After 24 h of incubation, 100 μL of supernatant from each well of the 96 well-microtitre plates were transferred into new 96-well microtitre plates and an equal volume of Griess reagent (Sigma Aldrich) was added. The absorbance of the mixture was determined at 550 nm on a microplate reader (Synergy Multi-Mode Reader, BioTek) after 10 min of incubation at room temperature. The quantity of nitrite was determined from a sodium nitrite standard curve. The percentage of NO inhibition was calculated based on the ability of each sample to inhibit nitric oxide production by RAW 264.7 macrophages compared with the control (cells treated with LPS without samples). Subsequently, the cell viability was determined using the MTT assay as described above.

### Statistical analysis

All experiments were performed in triplicate and the results are presented as mean ± SE (standard error) values. Statistical analysis was carried out with GraphPad Instat 3.0 software and Student-Newman-Keuls or Dunnett’s tests were used to determine *P*-values for the differences observed between tested samples and positive controls. Results were considered significantly different when *P* < 0.05.

## Results and discussion

### Phytochemical analysis

Phytochemical analysis is a qualitative assay which indicates the presence of groups of compounds in a sample through the formation of a precipitate or a color change. The phytochemical composition of the extracts determined using various techniques is represented in Table 1. It was found that the extract from the bark (BSP) had the highest extraction yield followed by the extract from leaves (LSP). These two extracts contained all the group of compounds analysed which are saponins, alkaloids, phenolics, flavonoids, terpenoids and tanins. On the contrary, phenolics and flavonoids were not detected in the extracts from roots (RSP) and fruits (FSP). In addition, saponins were not found in the extract RSP.

### Free radical inhibition of extracts

In the present study, two antioxidant assays which involved the measurement of color disappearance caused by free radicals such as DPPH and ABTS were used. These assays are typically based on the scavenging capacity of radicals which are converted into a colorless product. The degree of this discoloration corresponds to the amount of ABTS or DPPH that has been scavenged. The free radical scavenging potency of the extracts is presented in Table 2. As indicated in this table, the LSP extract had the best antioxidant capacity among all the extracts that were tested. With the inhibitory concentration (IC_50_) of 7.98 μg/mL and 15.35 μg/mL using the DPPH and ABTS assays respectively, this extract (LSP) was significantly (*P* < 0.05) more potent than all the extracts used. Differences in the antioxidant potency between the extracts from different parts of the plant may be due to the variation in their chemical composition. In fact, secondary metabolites are stored in various parts of plants and their concentration in each part varies with the exposition of the plant to the environment [18]. It was observed from the phytochemical screening as presented in Table 1 that the extracts from the leaves (LSP) and bark (BSP) of *S. pobeguinii* were rich in phenolic compounds and flavonoids while the two other extracts (roots and fruits) did not contain these phytochemicals. Pharmacologically, flavonoids and phenolic compounds, especially polyphenols, are responsible for antioxidant activity and correlation between these groups of compounds and their antioxidant capacity is well established [19, 20].

**Table 2:** Inhibitory concentration (IC_50_ in μg/mL) of extracts from roots, fruits, bark and leaves of *Sarcocephalus pobeguinii* and positive controls (trolox and ascorbic acid) or quercetin

Assay	IC_50_ in μg/mL		
	DPPH inhibition	ABTS inhibition	NO inhibition
RSP	854.65 ± 25.44	> 1000	> 100
FSP	234.48 ± 22.55	691.86 ± 33.41	84.43 ± 4.97
BSP	134.33 ± 1.02	400.08 ± 3.12	78.40 ± 3.47
LSP	7.98 ± 0.45^a^	15.35 ± 0.13^a^	46.09 ± 1.53^a^
Ascorbic acid	1.56 ± 0.57^b^	1.89 ± 0.36^b^	nd
Trolox	3.57 ± 0.26^b^	5.23 ± 0.98^b^	nd
Quercetin	nd	nd	5.07 ± 0.54^b^

*nd* not determined, *LSP* Methanol extract from leaves of *S. pobeguinii*, *RSP* Methanol extract from roots of *S. pobeguinii*, *FSP* CH_2_Cl_2_/MeOH (1:2) extract from fruits of *S. pobeguinii*, *BSP* Methanol extract from bark of *S. pobeguinii*

Data are presented as means of triplicate measurements ± standard error

^a^ significant difference between the extracts tested, (p ˂ 0.05)

^b^significant difference between the extracts and the positive controls, (p ˂ 0.05)

### Inhibitory activity of extracts on nitric oxide production

**Fig. 1:**
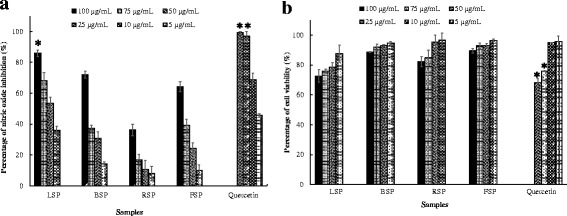
Nitric oxide inhibitory activity of the extracts from *S. pobeguinii* on RAW 264.7 macrophages (**a**) with respect to their cell viability (**b**). Data represent the mean ± SE (standard error) of three independent experiments, * means significantly different (p ˂ 0.05). LSP: Methanol extract from leaves of *S. pobeguinii*, RSP: Methanol extract from roots of *S. pobeguinii*, FSP: CH_2_Cl_2_/MeOH (1:2) extract from fruits of *S. pobeguinii*, BSP: Methanol extract from bark of *S. pobeguinii*

Nitric oxide (NO) is a pro-inflammatory mediator involved in various physiological events and its production is extremely important to defend the body. However, its overproduction can lead to tissue damage and activation of pro-inflammatory mediators associated with acute and chronic inflammation [21]. Therefore, NO inhibitory agents might be beneficial for treatment of the inflammatory response. In this study, the NO inhibition and cell viability were measured after treatment of RAW 264.7 macrophages with LPS and different concentrations of extracts, and quercetin as positive control (Fig. 1). From this figure, it is observed that the different extracts used in this experiment inhibited NO production in a concentration dependent manner. The highest significant (*P* < 0.05) NO inhibitory activity among the extracts was obtained with the LSP extract with a percentage inhibition of 85.87% at a concentration of 100 μg/mL (Fig. 1a). This extract (LSP) had also the lowest IC_50_ (46.09 μg/mL) which was significantly different (*P* < 0.05) from the other extracts as indicated in Table 2. The other extracts (BSP, RSP, FSP) were less effective than the LSP extract and this may be mainly attributed to the variation in their chemical composition as discussed in the previous paragraph. Quercetin, used as positive control, had the highest NO inhibitory activity with the percentage inhibition of 99.15% at 50 μg/mL (Fig. 1a) and IC_50_ of 5.07 μg/mL (Table 2). The viability of RAW 264.7 macrophages after treatment with the extracts and quercetin is presented in Fig. 1b. The extracts were slightly toxic against RAW 264.7 macrophages with percentage viability varying between 70 and 95% and interestingly, the LSP extract did not have significant cytotoxicity at the concentration leading to effective inhibition of NO production, although the percentage viability was lower compared to other concentrations tested (Fig. 1b).

### Antiproliferative activity of the extracts

**Fig. 2:**
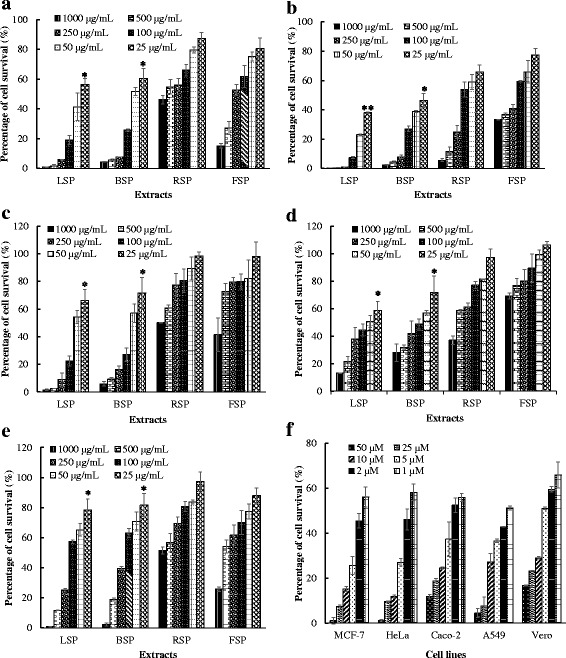
Antiproliferative activity of *Sarcocephalus pobeguinii* extracts against MCF-7 (**a**), HeLa (**b**), Caco-2 (**c**), A549 (**d**) and Vero (**e**) cell lines compared to the cytotoxic effect of doxorubicin on these cells lines (**f**). Data represent the mean ± SE (standard error) of three independent experiments. LSP: Methanol extract from leaves of *S. pobeguinii*, RSP: Methanol extract from roots of *S. pobeguinii*, FSP: CH_2_Cl_2_/MeOH (1:2) extract from fruits of *S. pobeguinii*, BSP: Methanol extract from bark of *S. pobeguinii*

The aim of this assay was to evaluate the inhibitory or cytotoxic activity of extracts on cancer cell proliferation and to compare their safety toward normal Vero cells. This goal is supported by the principle of selective toxicity which is the basis of cancer chemotherapy and the first choice of treatment for many cancers [22]. Figure 2 illustrates the antiproliferative activity of *S. pobeguinii* extracts against different cancer cell lines. It is observed from this figure that the percentage of cell survival varies in a concentration dependent manner with the LSP extract exhibiting the highest cytotoxic activity on all the cancer cell lines. Based on the results from Fig. 2, inhibitory concentrations (IC_50_) were calculated and are represented in Table 3. According to the National Cancer Institute (USA), an inhibitory concentration (IC_50_) of 30 μg/mL is the upper limit considered as promising for a crude extract [23]. Therefore, our results indicate that the LSP extract is active against human breast cancer cells (MCF-7) with IC_50_ of 26.94 μg/mL and human cervix cancer cells (HeLa) with IC_50_ of 10.19 μg/mL while the BSP extract is only efficient on HeLa cells with IC_50_ of 15.26 μg/mL (Table 3). The human cervix cancer cells (HeLa) were most susceptible with the lowest IC_50_ obtained for different extracts used. All the extracts were less toxic to normal Vero cells than to the four cancer cells except the extract from the fruits of *S. pobeguinii* (FSP) which was more toxic to Vero cells (LC_50_ of 601.42 μg/mL) than human epithelial colorectal cancer cells (IC_50_ of 721.03 μg/mL), which is statistically different (*P* < 0.05). The selectivity index (SI) varied between 0.83 and 18.28 and the LSP extract had the best SI ranging from 3.15 to 18.28 on the four cancer cells compared to the normal Vero cells. When compared with doxorubicin (SI between 1.61 and 4.61) which is commonly used in the treatment of leukaemia and Hodgkin’s lymphoma, as well as cancers of the bladder, breast, stomach, lung, ovaries, thyroid, soft tissue sarcoma, multiple myeloma, and others [24], the LSP extract had a higher selectivity index. Our results demonstrate that the LSP extract is selectively cytotoxic to cancer and not to normal cells, therefore suggesting the potential of this extract to be used as an antiproliferative agent. These results corroborate with those of Kuete et al. who reported the cytotoxic potential of leaves and bark of *Nauclea pobeguinii* against sensitive and multi-factorial drug-resistant cancer cell lines [13]. In addition to the cytotoxic activity of the extracts on cancer cells, the present study contributed in presenting the least toxic effect of the extracts against a non-cancerous cell and the selective cytotoxicity between cancer and non-cancerous cells which is the basis of cancer chemotherapy. The LSP and BSP extracts were very rich in alkaloids compared to the two other extracts. In fact, several alkaloids isolated from plants exhibit antiproliferative effects on various types of cancers, with compounds such as vincristine and vinblastine already having been successfully developed into anticancer drugs [25]. So, further investigations to identify the active compounds in *S. pobeguinii* are underway and may lead to the development of either new phytomedicines or pharmaceutical drugs against cancer.

**Table 3:** Cytotoxic activity (LC_50_ and IC_50_ in μg/mL) and the selectivity index (SI) of extracts from roots, fruits, bark and leaves of *Sarcocephalus pobeguinii* and reference drug (doxorubicin) against cancer cell lines

Cell lines	Vero	MCF-7		HeLa		Caco-2		A549	
	LC_50_ (μg/mL)	IC_50_ (μg/mL)	SI	IC_50_ (μg/mL)	SI	IC_50_ (μg/mL)	SI	IC_50_ (μg/mL)	SI
RSP	>1000	557.75 ± 60.35	nd	175.50 ± 31.37	nd	>1000	nd	857.25 ± 63.55	nd
FSP	601.42 ± 4.42	453.27 ± 6.41	1.32	194.81 ± 9.39	3.08	721.03 ± 68.35	0.83	>1000	nd
BSP	215.76 ± 10.83^a^	70.23 ± 0.99	3.07	15.26 ± 0.45^a^	14.13	62.65 ± 3.30^a^	3.44	92.21 ± 0.91	2.33
LSP	186.30 ± 5.42^a^	26.94 ± 1.15^a^	6.91	10.19 ± 0.31^a^	18.28	59.02 ± 2.37^a^	3.15	50.46 ± 1.65^a^	3.69
Doxorubicin (μM)	4.85 ± 0.29^b^	1.78 ± 0.39^b^	2.72	1.92 ± 0.29^b^	2.52	3.00 ± 0.24^b^	1.61	1.05 ± 0.67^b^	4.61

*nd* not determined, *LC*_*50*_ concentration which is lethal to 50% of the cells compared to untreated controls, *IC*_*50*_ concentration required to inhibit the cell growth by 50% compared to untreated controls, *LSP* Methanol extract from leaves of *S. pobeguinii*, *RSP* Methanol extract from roots of *S. pobeguinii*, *FSP* CH_2_Cl_2_/MeOH (1:2) extract from fruits of *S. pobeguinii*, *BSP* Methanol extract from bark of *S. pobeguinii*

Data are presented as means of triplicate measurements ± standard error

^a^ significant difference between the extracts tested, (p ˂ 0.05)

^b^ significant difference between the extracts and the positive control, (p ˂ 0.05)

### Correlation between free radical inhibition capacity and antiproliferative activity

The correlation analysis was done to understand the relationship between the scavenging capacity of extracts and their antiproliferative activity as indicated in Table 4. A positive linear correlation (0.4542 to 0.9914) was observed between free radical scavenging and antiproliferative activity, and the highest correlation was obtained with human epithelial lung cancer cells (A549).

**Table 4:** Correlation between radical inhibition potency of extracts from roots, fruits, bark and leaves of *Sarcocephalus pobeguinii* with respect to their antiproliferative activity against cancer cell lines

Cell lines	Correlation factor (R^2^)		
	DPPH inhibition	ABTS inhibition	NO inhibition
MCF-7	0.6923	0.7614	0.6879
HeLa	0.4542	0.7009	0.4544
Caco-2	0.6946	0.6830	0.3908
A549	0.9914	0.7206	0.9435

On the one hand, ABTS and DPPH are free radicals with the ability to accept an electron or hydrogen radical to yield stable molecules. Thus, the effects of antioxidants on DPPH or ABTS radical scavenging is thought to be due to their electron or hydrogen-donating ability [26]. The evaluation of the antioxidant activity of samples has been carried out using the DPPH and ABTS scavenging methods due to their simple, rapid, sensitive and reproducible procedures. Therefore, the radical scavenging assays in the cell-free systems for antioxidant studies are often considered by researchers before further studies in cell lines and/or animal models [27]. Our study suggested that the free radical scavenging capacity of extracts could contribute either moderately or strongly to their antiproliferative activity, which is supported by the fact that antioxidants known as “free radical scavengers” act by preventing and repairing damage caused by reactive oxygen species (ROS) and reactive nitrogen species (RNS), and thus can lower the risk of cancer [28, 29]. Other authors have reported a positive linear relationship between antioxidant activity and anticancer effect of five herbal water extracts by comparing their percentage free radical scavenging capacity and percentage growth inhibition on A549 and MCF-7 cells [30]. Similarly, the work of Chaudhary et al. showed that the extract and fractions of *Nardostachys jatamansi* exhibited both anticancer and antioxidant activities [31].

On the other hand, nitric oxide (NO), a short-lived free radical generated from L-arginine, is implicated in carcinogenesis and thus is closely related to cancer [32]. In fact, the longer the inflammation persists, the higher the risk of cancer. So, it makes sense that by inhibiting NO production using RAW 264.7 macrophage cells as a model, an extract might also exhibit anticancer activity. In this study, nitric oxide was considered as a free radical and a positive correlation (R^2^ varying between 0.3908 and 0.9435) was observed between NO inhibition and anticancer activity. Then, the anti-inflammatory activity might be considered as an additional beneficial property for extracts exhibiting anticancer activity since the inflammation process is closely related to the risk of cancer progression.

## Conclusions

The goal of our study was to evaluate the free radical scavenging potency of extracts from *Sarcocephalus pobeguinii* together with selective cytotoxicity on cancer cell lines compared to normal Vero cells. In this work, the extract from leaves of *S. pobeguinii* (LSP) exhibited the highest free radical inhibitory and antiproliferative activities, suggesting that the radical scavenging capacity of extracts might contribute to predict their anticancer properties. The high selectivity index of this extract indicates its potential as a source of drug discovery and development against cancer.

## Abbreviations

ABTS: 2,2′-azino-bis (3-ethylbenzothiazoline-6-sulfonic acid)
BSP: Methanol extract from bark of *Sarcocephalus pobeguinii*
DMSO: Dimethyl sulfoxide
DPPH: 2,2-diphenyl-1-picrylhydrazyl
FSP: CH_2_Cl_2_/MeOH (1:2) extract from fruits of *Sarcocephalus pobeguinii*
LPS: Lipopolysaccharide
LSP: Methanol extract from leaves of *Sarcocephalus pobeguinii*
MTT: Methyl thiazol tetrazolium
NO: Nitric oxide
RSP: Methanol extract from roots of *Sarcocephalus pobeguinii*
